# Phytic Acid and Whole Grains for Health Controversy

**DOI:** 10.3390/nu14010025

**Published:** 2021-12-22

**Authors:** Fred Brouns

**Affiliations:** Department of Human Biology, School for Nutrition and Translational Research in Metabolism, Maastricht University, 6200 MD Maastricht, The Netherlands; fred.brouns@maastrichtuniversity.nl

**Keywords:** phytic acid, phytate, mineral bioavailability, whole grain

## Abstract

Phytate (PA) serves as a phosphate storage molecule in cereals and other plant foods. In food and in the human body, PA has a high affinity to chelate Zn^2+^ and Fe^2+^, Mg^2+^, Ca^2+^, K^+^, Mn^2+^ and Cu^2+^. As a consequence, minerals chelated in PA are not bio-available, which is a concern for public health in conditions of poor food availability and low mineral intakes, ultimately leading to an impaired micronutrient status, growth, development and increased mortality. For low-income countries this has resulted in communications on how to reduce the content of PA in food, by appropriate at home food processing. However, claims that a reduction in PA in food by processing per definition leads to a measurable improvement in mineral status and that the consumption of grains rich in PA impairs mineral status requires nuance. Frequently observed decreases of PA and increases in soluble minerals in in vitro food digestion (increased bio-accessibility) are used to promote food benefits. However, these do not necessarily translate into an increased bioavailability and mineral status in vivo. In vitro essays have limitations, such as the absence of blood flow, hormonal responses, neural regulation, gut epithelium associated factors and the presence of microbiota, which mutually influence the in vivo effects and should be considered. In Western countries, increased consumption of whole grain foods is associated with improved health outcomes, which does not justify advice to refrain from grain-based foods because they contain PA. The present commentary aims to clarify these seemingly controversial aspects.

## 1. Backgrounds

A sufficient daily supply of minerals and trace-elements in the food that we consume, their subsequent exposure to digestion enzymes (bio-accessibility) and the quantitative absorption and supply to cells and organs (bio-availability) are crucial for our micronutrient status. A poor mineral status, most significantly of calcium, potassium, magnesium, zinc, iron and manganese are associated with an impaired immune function and elevated chronic disease risks. For example, iron deficiency leading to anemia is related to poor body functioning and increased morbidity and mortality in all age groups [1,2,3,4,5] The first months of life are particularly crucial because of the lasting effects later in life, even after iron stores restoration [6]. A poor magnesium status is associated with increased risks of diabetes and osteoporosis [7,8] and mineral-trace element deficiencies are related to impaired immune competence, especially in the elderly, and stunted growth in children [9,10,11]. Depending on country, food culture, food availability and related food purchases, the daily phytate intake is known to differ between westernized and low-income countries such as south and southeast Asia and central Africa, as well as between urban (large cities) and rural Chinese communities, where many low-income families reside [12,13]. These families largely rely on ‘monotonous’ consumption, mostly of one particular crop rich in PA, such as rice or corn [14,15], often resulting in mineral deficiency and related impaired growth and health. The reason for this is that the amount of phytate bound minerals, excreted in fecal matter, often exceeds the total mineral amount ingested, leading to a net negative balance. The fact that the presence of PA during the entire intestinal transit also chelates minerals and trace-elements that are part of fluxes between blood and gut is of influence. The recognition of mineral deficiency in large populations and the related cause-effects has led to the development of a number of strategies to reduce the phytates content in food during food processing, with the aim to enhance mineral bio-availability [16,17,18,19,20]. The following sections will provide a brief introduction to the molecular aspects of PA, phytase and the presence of PA in various grains and bread.

### Data Search Supporting this Narrative Critical Commentary

For this critical commentary, available data (period 1990-today) were searched for in common electronic databases such as PubMed, ScienceDirect, Web of Science, and other connected data bases at the Maastricht university online library. Searches were performed for the key words phytic acid, phytate, phytase, in combination with digestion, bio-accessibility, bio-availability minerals, trace-elements, sourdough, yeast, bread making, food processing, fermentation, microbial metabolism, health effects, micronutrient deficiencies, immune, chronic disease, hidden hunger and stunted growth. In addition, reference listing of selected publications was screened for additional original data sources. Inclusion criteria were as follows: peer reviewed papers in English language: original research, critical reviews and commentaries.

## 2. Phytic Acid and Phytate

Phytic acid (PA) is a six-fold dihydrogenphospate ester of inositol also referred to as (myo-inositol 1,2,3,4,5,6 hexakisphosphate). Numbers 1–6 stand for the presence of 6 potential binding sites. PA serves as a phosphate storage (Ins P6) molecule in cereals, which, due to its affinity for metal ions, also strongly chelates the cations Zn^2+^ and Fe^2+^, as well as Mg^2+^, Ca^2+^, K^+^, Mn^2+^ and Cu^2+^. During seed development, PA accumulates as mixed salts containing P, Mg and K and to a lesser extent Ca, Zn and Mn [20,21]. Studies in humans and animals, utilizing various crops differing in PA content, show a more or less linear increase in iron and zinc bioavailability with decreasing PA [20], which is due to the linear decrease in overall binding sites with decreasing PA content.

Because of the absence of endophytases, phytate cannot be degraded by the human body, and cannot be absorbed in the small intestine [22]. As a consequence, the minerals chelated in PA are not bio-available. However, PA mineral bonds can be reversed by phytase-induced degradation of PA during food processing (by means of phytases present in the plant/flour) as well as during digestion (by phytase activity expressed in microbiota residing in the intestinal tract). Apart from being a mineral chelating molecule, PA can also influence amino-acid and carbohydrate metabolism (by binding of amino acids, peptides and digestion enzymes), act as a strong antioxidant (e.g., reducing iron induced lipid peroxidation) and express anticancer properties (e.g., by binding the strong pro-oxidant free iron and reinforcing apoptosis in cancerous cells). Sodium phytate is registered as a generally recognized safe substance that can be used for food stability in a wide variety of applications [23]. Overall, a wide range of favorable and less desired properties has been reported [20,24,25,26,27]. Although precise mechanisms are often unclarified. Many of the suggested PA benefits are based on in vitro studies, directly exposing cells to PA, whereas in vivo PA cannot be absorbed when fully intact and needs to be degraded by phytases, after which only lower inositol phosphates or the inositol back bone may pass into the circulation and express bio-activity.

## 3. Phytic Acid and Phytate, Good or Bad for Health?

At present there is increased marketing for fermented foods, such as bread produced with sourdough processing, resulting on low PA levels in dough and bread. Seen the increasing international recommendations to consume more whole grain foods (potentially high in PA) for better health, and the voices that state “do not, it contains phytate” there is a need to put this potential controversy in perspective of the overall dietary and population conditions.

In the grain kernel, the bran is rich in phytate. Environmental conditions of growth, most importantly the soil mineral content, plays a significant role in crop mineral content, which also interacts with PA content [18,28]. The contents of these depend significantly on the type of grain (in order of decreasing magnitude of the ‘highest in range’ values: sorghum > wild rice > maize > rye, wheat > oat, barley [24,26,29]. The endogenous phytase contents also differ among crops. With respect to the overall diet, it should be noticed that various legumes and seeds, such as sesame, linseeds, kidney beans, maize and soy generally have higher PA contents, compared to the most consumed cereal bread-wheat. An extensive overview of PA contents in food can be found in Schlemmer et al. 2009 [24]. Thus, although whole-grain consumption supplies phytates to our body, it is only one of many sources. During milling, bran and germ can be separated from the endosperm and the resulting white flour is consequently very low in phytate and minerals, as are all other ‘refined’ cereal products.

## 4. Phytates in Bread

Over the last decade, the baking industry has intensified attempts to reduce PA in whole grain bread, using two potential approaches: (1) inducing acidity during dough fermentation in bread making, which increases endogenous phytase activity, and by (2) adding exogenous phytases to the dough making process.

Ad 1: Studies show that the type of grain (wheat, rye, oat, maize, etc., having different PA and endogenous phytase contents), the type of flour used (whole meal, white flour) and the fermentation conditions (yeast or sourdough (SD), their specific microbiota composition, fermentation duration and temperature, level of pH reduction) are mutual determinants of phytase activity and phytate degradation in the dough [30,31,32,33]. Due to the high content of PA and phytates in the bran fraction of whole grain, compared to isolated white flour, SD ferments of whole grain flours, resulting in significant decreased dough pH, show the highest phytate reductions and related increase in free Ca^2+^, Zn^2+^, Fe^2+^, Mg^2+^ [34]. Long-term SD fermentation, with a mutual effect of acidification (which stimulates flour endogenous phytases) and the presence of phytates expressing microbiota, results in significantly higher PA degradation, compared to short term yeast fermentation. The latter is due to less acid formation and the low phytase activity of yeast [35,36]. Studies have been conducted to evaluate the effect of specific strains of lactobacillus, bifidobacteria and selected baker’s yeast strains of the extent of PA degradation during the dough making, e.g., [15,37,38,39,40,41,42,43].

However, the benefit of the need for use of selected lactobacilli for this purpose is challenged by data from Leenhardt et al. [44], who showed that only moderately acidifying the dough with lactic acid (not less than pH 5.5), in the absence of lactic acid bacteria, resulted in similar PA degradation compared to the use SD fermentation alone. This data indicates that only moderate acid content is required to enhance endogenous phytase activity and mineral bio-accessibility of the dough and resulting bread.

Ad 2: The addition of exogenous phytases during dough processing, such as Aspergillus Niger, have the potential of PA degradation to levels of 100% [40].

## 5. Limitations of Approaches and Data

Generally, in vitro digestion studies confirm that fermentation-induced acidification (SD fermentation > yeast fermentation) of the dough reduces the phytate content and increases the content of free ionized minerals, in favor of better bio-accessibility [17,31,33,35,43,45,46].

Yet, an improved bio-accessibility, which potentially facilitates the bio-availability in the small intestine of Zn^2+^, Fe^2+^, Mg^2+^, Ca^2+^, does not necessarily result in an improved mineral status of the human body. The latter is the longer-term effect of digestion and the absorption of multiple meals passing the entire gastrointestinal tract, and the subsequent distribution and excretion of absorbed/non absorbed minerals. This encompasses much more than the digestion of a single test meal in vitro in a simulated intestinal compartment, where many in vivo factors (neural regulation, blood flow, hormonal responses, gut epithelium associated factors, microbiota metabolism, etc.) are absent. The mutual presence of many mineral bio-accessibility and bio-availability inhibiting and promoting factors in the diet, and in the entire intestinal tract, plays a crucial role in overall mineral absorption. In this regard, Cook et al. [47] showed that single-meal studies may overemphasize the influence of enhancers and inhibitors on iron absorption, in comparison to multi-meal studies, due to other factors not present in the test conditions.

In addition, after the initial ingestion of bread, the phytates that are still present (non-degraded during dough fermentation as measured in an in vitro essay) may be partially broken down due to phytase activity induced by acid exposure in the stomach. Moreover, in the colon, where a pH reduction can occur due to the fermentation of dietary fibers and where specific microbiota species with a high phytase activity are present, significant PA degradation can take place, resulting in the solubilization of mineral complexes, an increase in ionized free minerals and their colonic absorption. The presence of fecal matter in the colon for a long period, combined with the high microbial phytase activity make the colon the prime PA degrading area in the human body. As a consequence, the remaining PA in fecal matter is extremely low [24,48]. Thus, a complete picture of bio-availability and its impact on the mineral status of cells, organs and bone, resulting from the entire gastrointestinal tract in humans, can only be obtained by performing complex net balance studies and mineral density measures in vivo, which are laborious and expensive.

### 5.1. In Vitro versus In Vivo Testing

#### 5.1.1. In Vitro

Nevertheless, and because of costs and practicality, the in vitro digestion-absorption assays are being used at an increasing rate to predict what potentially may happen in vivo, and, over the last decade, numerous studies have been performed addressing the effects of dough fermentation on in vitro digestibility and bio-accessibility, which is not a measure of bioavailability. To identify whether the observed increase in free minerals measured during in vitro digestion enhances intestinal absorption, an additional assay is being used, in which the obtained digestion effluent is exposed to in vitro cultured intestinal cells (usually Caco-2 cells), followed by measuring the uptake of selected minerals by these cells. Rodriguez Ramiro et al. [35] showed that the fermentation induced decrease in phytate and the increase in ionized minerals (bio-accessibility) cannot be directly generalized to better absorption/bio-availability. They confirmed that whole-grain SD bread had a greater phytate degradation than yeast bread (resp. SD 100% vs. yeast 75%). However, neither the yeast nor the SD bread resulted in increased ferritin levels (a measure of iron uptake) in the Caco-2 cells. These findings were in agreement with others [49] who also reported a significantly increase in bio-accessible iron as a result of fermentation but a negligible effect on iron absorption in Caco-2 cells. Thus, in the Caco-2 cells test, other factors appear to influence the step between bio-accessibility and bio-availability and ferulic acid has been suggested as one example [50,51,52].

#### 5.1.2. In Vivo May Differ from In Vitro

The results in vivo can differ from those obtained in vitro. An increase in soluble minerals in vitro does not necessarily translate into an increase in bioavailability in vivo because other environmental/dietary factors may play a role. For example, Hoppe et al. [53] observed that the consumption of a low-phytate wholegrain bread in a total diet context, compared to a high-phytate wholegrain bread, did not improve iron status in humans. Others observed no significant effects in iron absorption from meals prepared using genetically modified maize containing 30–50% less phytic acid, compared to regular maize [54,55]. Low PA cereal porridges prepared in water, often consumed in low-income countries, contain higher levels of Fe^2+^ and promote iron absorption, but this effect appears to be absent when the porridges were prepared with milk, most likely due to the presence of calcium and casein [56,57]. The presence of vitamin C may significantly enhance iron absorption, although this is not always the case [58], and a significant intake of fermentable dietary fibers may reduce the colonic pH due to microbial fermentation, leading to the solubilization of unabsorbed mineral complexes and a subsequent absorption of the free minerals [59]. Studies on the effects of phytate, phytase and fiber addition on apparent total intestinal tract digestibility of Ca and P in pigs found that the presence of fiber increased digestibility and bio-availability [48,60]. On the other hand, the medically prescribed use of proton-pump inhibitors to reduce gastric acid production and reflux, is known to be associated with calcium, iron and magnesium deficiencies, likely due to effects of reducing PA degradation and mineral solubilization [61]. Thus, PA is only one of many factors influencing overall mineral uptake by the human body and the reduction of PA levels in food alone, without consideration of other factors, may slightly increase total bio-accessibility but may be ineffective in reducing the prevalence of mineral deficiencies.

In line with this reasoning, studies have shown that strategies to help reduce the PA content in food during food preparation in low-income countries may be insufficient to significantly counterbalance low iron and zinc levels in children. It was shown that a parallel increase in the consumption of meat and other non-PA food sources, relatively rich in iron and zinc, are required to fully overcome this problem [62,63]. Alternatively, the mineral fortification of food may be of added value. Increasing the total iron content of the bread by exogenous iron fortification resulted in a significant increased Fe^2+^ uptake in Caco-2 cells [35] and consuming iron enriched SD bread, but not plain SD bread, improved iron status [45]. A recent meta-analysis of studies concluded that flour fortification with iron appears to be a very effective public health strategy for the improvement of iron/mineral status globally [5].

## 6. Concluding Remarks

An appropriate micronutrient status has a significant impact on health and is compromised in situations of overall marginal food and nutrient intakes, as is evident in low- income countries [64,65].

The available evidence clearly indicates that, for the enhancement of iron absorption in individuals who have a marginal mineral intake, the fortification of flour (foods) is more effective than fermentation-induced PA degradation during food processing alone. Although fermentation conditions vary widely, and the fortification of flours can be realized with high precision and within narrow ranges. A combination of fermentation and fortification may nevertheless potentiate positive effects. The fact that a lower PA will offset the potential PA-induced effects on certain health parameters is still challenging [20].

In Western countries, it is increasingly recommended to consume a diet rich in whole grains, legumes, vegetables, seeds and nuts, which seems controversial since most of these are relatively high in PA. However, there is no doubt that this is associated with improved health outcomes [53,66,67,68,69,70,71,72].

The advice to avoid the consumption of whole grain foods because they contain PA is unjustified. 
